# Venous thrombosis and obesity: from clinical needs to therapeutic challenges

**DOI:** 10.1007/s11739-024-03765-7

**Published:** 2024-09-13

**Authors:** Federica La Rosa, Fabrizio Montecucco, Luca Liberale, Marta Sessarego, Federico Carbone

**Affiliations:** 1 First Clinic of Internal Medicine, Department of Internal Medicine, University of Genoa, 6 Viale Benedetto XV, 16132 Genoa, Italy; 2 IRCCS Ospedale Policlinico San Martino, Genoa - Italian Cardiovascular Network, Genoa, Italy

**Keywords:** Obesity, Thrombosis, Venous thromboembolism, Body mass index, Cardiovascular

## Abstract

Weight bias and stigma have limited the awareness of the systemic consequences related to obesity. As the narrative evolves, obesity is emerging as a driver and enhancer of many pathological conditions. Among these, the risk of venous thromboembolism (VTE) is a critical concern linked to obesity, ranking as the third most common cardiovascular condition. Obesity is recognized as a multifactorial risk factor for VTE, influenced by genetic, demographic, behavioral, and socio-economic conditions. Despite established links, the exact incidence of obesity related VTE in the general population remains largely unknown. The complexity of distinguishing between provoked and unprovoked VTE, coupled with gaps in obesity definition and assessment still complicates a tailored risk assessment of VTE risk. Obesity reactivity, hypercoagulability, and endothelial dysfunction are driven by the so-called ‘adiposopathy’. This state of chronic inflammation and metabolic disturbance amplifies thrombin generation and alters endothelial function, promoting a pro-thrombotic environment. Additionally, the inflammation-induced clot formation—also referred to as ‘immunothrombosis’ further exacerbates VTE risk in people living with obesity. Furthermore, current evidence highlights significant gaps in the management of obesity related VTE, particularly concerning prophylaxis and treatment efficacy of anticoagulants in people living with obesity. This review underscores the need for tailored therapeutic approaches and well-designed clinical trials to address the unique challenges posed by obesity in VTE prevention and management. Advanced research and innovative strategies are imperative to improve outcomes and reduce the burden of VTE in people living with obesity.

## Introduction

Weight bias and stigma have affected people living with obesity and their lifespan for decades [1]. The increasing awareness of the obesity pandemic is now changing its narrative and now recognizes different and specific clinical needs to be addressed. Obesity is a complex and heterogeneous disease associated with important health issues, not limited to the cardiovascular (CV) system. Dealing with them is becoming a medical subspecialty requiring new knowledge and different approaches [2]. Among these issues, venous thromboembolism (VTE), including deep vein thrombosis (DVT) and pulmonary embolism (PE), is of epidemiological relevance. As the third most common CV disease, VTE carries with it a substantial burden of death—around 20%—and short- and long-term complications related to both disease (e.g., recurrence, post-thrombotic syndrome) and treatment (i.e., major bleeding associated with anticoagulation treatment) [3, 4]. Obesity is among the risk factors for VTE and contributes to its growing incidence linearly with increasing body mass index [5]. Obesity also encompasses a wide range of genetic, demographic behavioral and socio-economic conditions that further enhance the risk of VTE such as sedentariness, discrimination, limited access to healthcare [6]. Despite evidence of a causal relationship [7, 8], the incidence proportion of VTE related to the obesity in the general population is not clearly defined. Furthermore, clot biology changes between provoked and unprovoked VTE, a sometime elusive distinction that likely underestimates individual risk. While the link between obesity and thrombosis is long-time established on the arterial front, less is known on why and how VTE occurs more frequently in people living with obesity. To address those questions is of paramount importance as their impact is not limited to the risk of VTE but also concerns its recurrence and risk related to blood thinners. This narrative review has been then designed to update the current knowledge of VTE applied to people living with obesity, spanning from their increased risk to the issue related to anticoagulant therapy.

## What is known and what is new about the link between obesity, platelet activity, and hypercoagulability: lessons of atherothrombosis

Whatever defined, obesity represents an established risk factor for arterial thrombosis, both in coronary and peripheral settings. In the mainstream view of metabolic syndrome (MetS), clustering obesity with other established cardiovascular risk factors would confer a synergistic atherothrombotic risk that encompasses plaque development, progression, and vulnerability until clot formation and density [9]. Large studies and meta-analyses clearly address the atherothrombotic risk related to obesity [10–14] and emphasize the benefits of tackling it [15–18]. With the rise of the ‘residual cardiovascular risk’ paradigm, obesity has become a risk factor in its own right. The pathogenic potential of excess body fat is indeed conditioned on its visceral extent and dysfunction, the latter defined by inflammation, insulin resistance, and ectopic fat deposition [19]. Those central tenets—also known as ‘adiposopathy’—are all promoters of platelet hyperactivity/dysfunction and an hypercoagulation state.

### Platelet activity

Platelet reactivity indeed increases with the extent of visceral adiposity [9, 20–24]. All central tenets of adiposopathy contribute to this effect by increasing cytosolic Ca^2+^ concentrations, the production of thromboxane A2 (TxA2) from arachidonic acid [25], and the surface expression of P-selectin and glycoprotein IIb/IIIa, along with a greater resistance to nitric oxide (NO) and prostaglandin I2 [26, 27]. Especially, insulin resistance seems implicated platelet hyper-reactivity, whereas weight loss/improvement of insulin sensitivity have direct effects on the splicing of tissue factor (TF). Additional effects on platelet have been described for hypertriglyceridemia and low levels of high-density lipoproteins [28]. The excess of lipid peroxidation upregulates the generation of TxA2 and stimulates platelet aggregation. Adipokines imbalance (i.e., adiponectin/leptin ratio) and pro-inflammatory environment directly alter platelet function as well. They are associated with high surface expression and circulating levels of the CD40 ligand. These effects may also extend beyond circulating platelet and affect their transcriptome directly within megakaryocytes [29–33]. Not surprisingly, weight loss has recently been hypothesized as modifier of platelet count and function [34–36].

### Hypercoagulation state

Furthermore, the atherothrombotic risk associated with obesity and insulin resistance is linked to a hypercoagulable state. The thrombin generation potential significantly increases with visceral adiposity and insulin resistance, while it decreases with weight loss in people with severe obesity and with treatment using metformin [21, 37–39]. The up-regulation of the TF pathway has been widely observed because of higher plasma concentrations of FVII and increased levels of thrombin and thrombin–antithrombin (TAT) complexes. Several hormonal and metabolic changes characterizing obesity and insulin resistance are known modifiers of TF expression and activity. In the local microenvironment of dysfunctional visceral adipose tissue (VAT), macrophage expression of TF—under the control of the toll-like receptor-nuclear factor-κB (NF-κB)/Jun N-terminal kinase (JNK) cascade—is upregulated by lipolysis, imbalance of adiponectin/leptin (i.e., low adiponectin and/or high leptin), and insulin resistance (this latter via phosphatidylinositol 3-kinase [PI3K]) [40–43]. In turn, TF even contributes to the pathophysiology of obesity through coagulation-independent mechanisms. Engineered mice lacking the TF cytoplasmic domain experience slower weight gain, increased energy expenditure, and improved glucose homeostasis [44]. Similarly, inhibiting the TF-FVIIa binding rapidly improves metabolism and fatty acid oxidation [45]. TF is also a known enhancer of adipose tissue inflammation and insulin resistance. Within myeloid cells TF signaling contributes to the recruitment and/or retention of pro-inflammatory adipose tissue macrophages (ATMs) and their activation (i.e., polarization toward the pro-inflammatory M1 phenotype) [21]. Specific deletion of TF cytoplasmic tail in murine myeloid cells indeed suppresses the production of pro-inflammatory molecules in response to a high-fat diet [44]. These changes have a limited effect on weight gain but markedly improve insulin resistance and hepatic steatosis. In this scenario, adipocyte–macrophage interactions seem necessary to activate pro-thrombotic responses in people living with obesity. While TF typically remains in a non-coagulant state within myeloid cells, it is rapidly activated via the P2X7 receptor in response to ATP and/or free fatty acids. As both ATP and free fatty acids are abundant in VAT, this environment may be considered a source and repository for ‘pro-coagulant ATMs’, enhancers of the overall thrombotic risk [46]. Levels of fibrinogen, von Willebrand factor (vWF), and factors VII and VIII are also increased in patients living with obesity. While extravascular fibrin deposits are found in liver and dysfunctional VAT [47], higher plasma activity of clotting factors VII and VIII as well as increased plasma levels of fibrinogen and von Willebrand factor antigen are commonly found in patients with type 2 diabetes [48]. Beside the hypercoagulation state, dysfunctional VAT also associates with disturbances in the fibrinolytic system. Due to the increased release of PAI-1 and the thrombin activatable fibrinolysis inhibitor from liver and dysfunctional VAT, high-fat diet and obesity inhibit plasminogen activators (PAs) (e.g., t-PA and urokinase-type plasminogen activator) thus preventing clot dissolution [48, 49].

## Obesity from athero- to venous thrombosis: the need for a pathophysiological reappraisal

Just as atherothrombosis extends beyond platelet involvement, VTE is not merely a coagulation-dependent process. The current understanding suggests intertwined mechanisms across both sides of the vascular system [50]. From the seminal meta-analysis by Ageno and co-workers, a large body of clinical evidence now supports a link between VTE and traditional cardiovascular risk factors with a prominent role of ‘adiposopathy’ (Table 1) [51–60].

**Table 1:** List of studies summarizing the risk of venous thromboembolism according with established cardiovascular risk factor

Author	Year	Study design and patients		Outcome	Results
Ageno W et al. [51]	2007	Meta-analysis of 21 prospective studies (*n* = 63,552) Aged < 55 years (n. of studies = 6), > 45 y (*n* = 4) or ranging 42–70 years (*n* = 11) Median FU 5.6 to 26y Both sexes (*n* = 10), only women (*n* = 8) or men (*n* = 3)	BMI addressed in 15 studies (*n* = 34,803) OB (prevalence 8.3% in cases *vs.*3.6% in controls) Hypertension DM Smoking Hypercholesterolemia	VTE (*n* = 31,138/3665)	The risk of VTE was 2.33 for obesity (95% CI 1.68–3.24), 1.51 for hypertension (95% CI 1.23–1.85), 1.42 for DM (95% CI, 1.12–1.77) but not significant for smoking and hypercholesterolemia. However, when lower-quality case–control studies were excluded, there was not any statistical heterogeneity
Linnemann B. et al. [52] Luxembourg B. et al. [53]	2008 2009	Case–control from MAISTHRO database (*n* = 1006) Mean age 43.5 years Median FU 40 months Men/women 424/582	OB (prevalence 22.7%) Male sex Hypertension	VTE recurrence after anticoagulant discontinuation for a first episode (*n* = 324) Predictive role of fibrinogen, FVIII, and hs-CRP	OB did not significantly increase the risk of VTE recurrence (RR 1.1 [95% CI 0.85–1.42]) as for male sex (HR 1.7 [1.4–21.9]) and hypertension (HR 1.4 [1.05–1.78]) BMI was higher in unprovoked VTE and associated with high circulating fibrinogen (*p* < 0.001)
Tufano A et al. [54]	2008	Cross-sectional trial (FAST) (*n* = 15,180) Aged ≥ 18 years Men/women 37.2%/62.8%	OB DM	Prevalence of recent VTE (< 1y) (*n* = 552)	OB was the higher prevalent risk factor for recent VTE in man (OR 1.97 [95% CI 1.40–2.78]) and the second one in women (OR 2.29 [95% CI 1.85–8.84]). A cumulative effect of multiple risk factors detected a multiplicative fashion for OB and diabetes
Katz Marcelo et al. [55]	2014	Case control from the NAVIGATOR trial (*n* = 9306) Aged ≥ 55 years or ≥ 55 years with established CVD men/women 4595/4711	BMI (median 29.7 kg/m^2^) WC (median 100 cm)	VTE risk in patients with IGT (*n* = 129)	Both WC and BMI were higher in the VTE group (*p* < 0.001)
Mahmoodi BK et al. [56]	2017	Meta-analysis of 9 prospective studies (*n* = 244,865) Mean age 49–73y FU 4.7–19.7y Both sexes (*n* = 8), only women (*n* = 1)	BMI (mean 25.2–29.1 kg/m^2^) Hypertension DM Dyslipidemia	VTE risk (*n* = 4910)	Although limited to adjustment variable, BMI (both categorized and continuous) overcame the predictive ability of other established CV risk factors like hypertension, diabetes, and dyslipidemia
Gaertner S et al. [57]	2018	Case–control data from REMOTEV registry (*n* = 515) Mean age 64 years FU 6 months Man prevalence 47.4%	OW prevalence 36.9% OB prevalence 33.2 DM Age	Provoked/unprovoked VTE (*n* = 190/325)	While hypertension (OR 1.44, [95% CI 1.01–2.06]), diabetes (OR 2.07, [95% CI: 1.25–3.55]), and age (OR 1.94, [95% CI: 1.31–2.88]) increase the risk of unprovoked VTE, only the association with OB remained significant at adjusted analysis (OR 1.8 [95%CI 1.07–3.18])
Gregson J. et al. [58]	2019	Pooled cohort studies from ERFC and UK Biobank (*n* = 1,135,124; 731,728 and 421,537) mean age 52 years and 56 years FU 15.4 years and 6.1 years Man prevalence 44.6%	BMI (mean 25.4/27.2 kg/m^2^) WC (88/90 cm) WHR (0.85/0.087) Age	VTE (*n* = 1041/2321)	Markers of adiposity were independent predictor of VTE in both ERFC and UK Biobank. HRs per 1-SD increase in BMI were 1.43 (95% CI, 1.35–1.50) in ERFC and 1.37 (95% CI, 1.32–1.41) in UK Biobank; for WC, the HRs were 1.60 (1.52–1.68) and 1.54 (1.37–1.73) and for WHR HRs were 1.52 (1.40–1.68) and 1.23 (1.05–1.45) Besides, age predicts VTE with HRs for decade of 2.67 (2.45–2.91) in ERFC and 1.81 (1.71–1.92) in UK Biobank
Mac Donald CJ et al. [59]	2021	Prospective from E3N cohort study set up in 1990 (*n* = 91,707 women) Mean age 51 years FU 1992 and 2005	BMI (mean 22.9 kg/m^2^) OW prevalence 16.4% OB prevalence 3.8% Hypertension Dyslipidemia DM Smoking	VTE risk (*n* = 1443)	As expected, CV risk increased during follow-up alongside with BMI (from 22.9 to 23.8 kg/m^2^). Although limited to adjustment variable, BMI overcame the predictive ability of other established CV risk factors like hypertension, dyslipidemia, diabetes and smoking
Wang H. et al. [60]	2022	AT-AGE case- control (*n* = 832) All aged ≥ 70y Man = 375	BMI (mean 27 kg/m^2^) OW prevalence 41.4/40.6% OB prevalence 21.9/21.8% Weight Smoking Alcohol DM Hypertension	VTE risk	VTE risk increased with weight (OR 1.5 [95% CI 1.0–2.4] and height but not BMI. While smoking, alcohol intake, and diabetes were not associated with VTE, hypertension was associated with a decreased risk of VTE

PubMed search (venous thromboembolism [Ti]) AND (cardiovascular risk [Ti])

*FU* follow/up, *BMI* body mass index, *DM* diabetes mellitus, *VTE* venous thromboembolism, *OB* obesity (defined by BMI values ≥ 30 kg/m^2^), *OR* odds ratio, *CI* confidence interval, *hs-CRP* high-sensitivity C-reactive protein, *RR* relative risk, *WC* waist circumference, *CVD* cardiovascular disease, *CV* cardiovascular, *OW* overweight (defined by BMI values of 25–29.9 kg/m^2^), *WHR* waist-to-hip ratio, *SD* standard deviation, *HR* hazard ratio

While Virchow’s triad still provides a conceptual framework, it is continuously reappraised [61]. Besides the above-mentioned hypercoagulation state, both endothelial injury/dysfunction and blood flow stasis are now joined by inflammation in the so-called ‘immunothrombosis phenomenon’.

### Blood flow stasis and endothelial injury

In the absence of any triggers, reduced and/or perturbed blood flow becomes the leading cause of VTE. Flow stasis may indeed overcome several regulatory circuits and trigger a signaling cascade converging on endothelial cell (EC) activation. The release and function of coagulation and fibrinolytic factors (e.g., thrombomodulin, protein C and protein S, thrombin and antithrombin activation, tissue factor pathway inhibitor, platelet activation and adhesion) are under endothelial control, which is highly dependent on NO production. When homeostasis is lost, NO concentrations fall, and ECs activate. As an endothelium-coated elastic vein valve model of DVT recently displayed, the release of vWF from Weibel–Palade bodies, critically contributes to the DVT initiation though an interaction with the GPIbα on platelet surface [62, 63]. This mechanism couples with an increased endothelial expression of adhesion molecules [64, 65] and both intersect with a dysregulated pro-inflammatory cascade.

Besides the increase in intra-abdominal pressure and reduction of venous blood flow velocity induce the VAT excess [66], the extent of blood flow perturbation extends beyond a ‘mechanical’ contribution to VTE. While the unidirectional laminar flow is detected by mechanoreceptors on ECs (e.g., cilia, glycocalyx, membrane receptors, and ion channels) and sustains elevated intracellular calcium and NO production via nuclear factor-E2-related factor 2 (Nrf2), this effect is lost in flow stasis [67, 68]. In such conditions, Nrf2 is suppressed alongside with cytoprotective enzymes, including heme-oxygenase 1 (HO-1) and superoxide dismutase (SOD). In this pro-oxidant microenvironment, the oxidation of thrombomodulin reduces its anticoagulant activity, whereas that of fibrinogens favors its conversion to fibrin, thus reducing its interaction with the anti-coagulant system [69, 70]. Alongside, a pathway-specific impairment in PI3K-dependent signaling characterizes insulin resistance and has long-time known to cause an imbalance between NO production and secretion of endothelin-1 [71]. Even leptin would be involved in the control of vascular tone by a somehow paradoxical simultaneous release of NO opposing to a neurogenic pressor action [72].

However, the pathophysiology of endothelial dysfunction in obesity now recognizes an ever-broader number of causes. Single cell RNA-sequencing profiling is unraveling the role of ECs in the homeostasis of adipose tissue and how deep the changes are during the development of obesity [73–75]. When focused on VAT-restricted mice ECs, single nucleus RNA transcriptomic has recently displayed their lower expansion coupled with a differential gene expression that modify with obesity severity. Especially, *hif1a* and *vegf* emerge as critically modified genes, hallmarks of inflammatory, angiogenic response and ultimately thrombotic risk [75].

Outside the microenvironment of VAT, clinical interest in endothelial microvesicles (EMVs) has recently intensified given their link with obesity and the putative role in hemostasis and thrombosis [76]. EMVs are indeed released in response to pro-thrombotic stimuli and exert themselves pro-thrombotic activities in both arterial and venous system. Once shed by activated/apoptotic cells (i.e., ECs but also platelet, neutrophil, monocyte, erythrocytes, and cancer cells), MVs contribute to the coagulation cascade through the exposure of phosphatidylserines, which promotes the assembly of tenase (factors VIIIa, IXa, X) and pro-thrombinase (factors Va, Xa, thrombin) complexes and directly associates with factor XII, thus supporting thrombin generation via activation of the intrinsic coagulation cascade. Furthermore, TF and its inhibitor (tissue factor pathway inhibitor) and the P-selectin glycoprotein ligand-1 (PSGL-1, CD162) are other pro-coagulant molecules/receptors carried by EMVs, enabling thrombus propagation. EMVs account for about 10% of physiologically circulating MVs but they have a high pro-coagulant potential at thrombodynamic test assay [77]. Circulating EMVs increase with obesity and modify their composition with negative effects on EC inflammation, viability, NO production, and pro-coagulant status [78, 79].

### Immunothrombosis

**Fig. 1:**
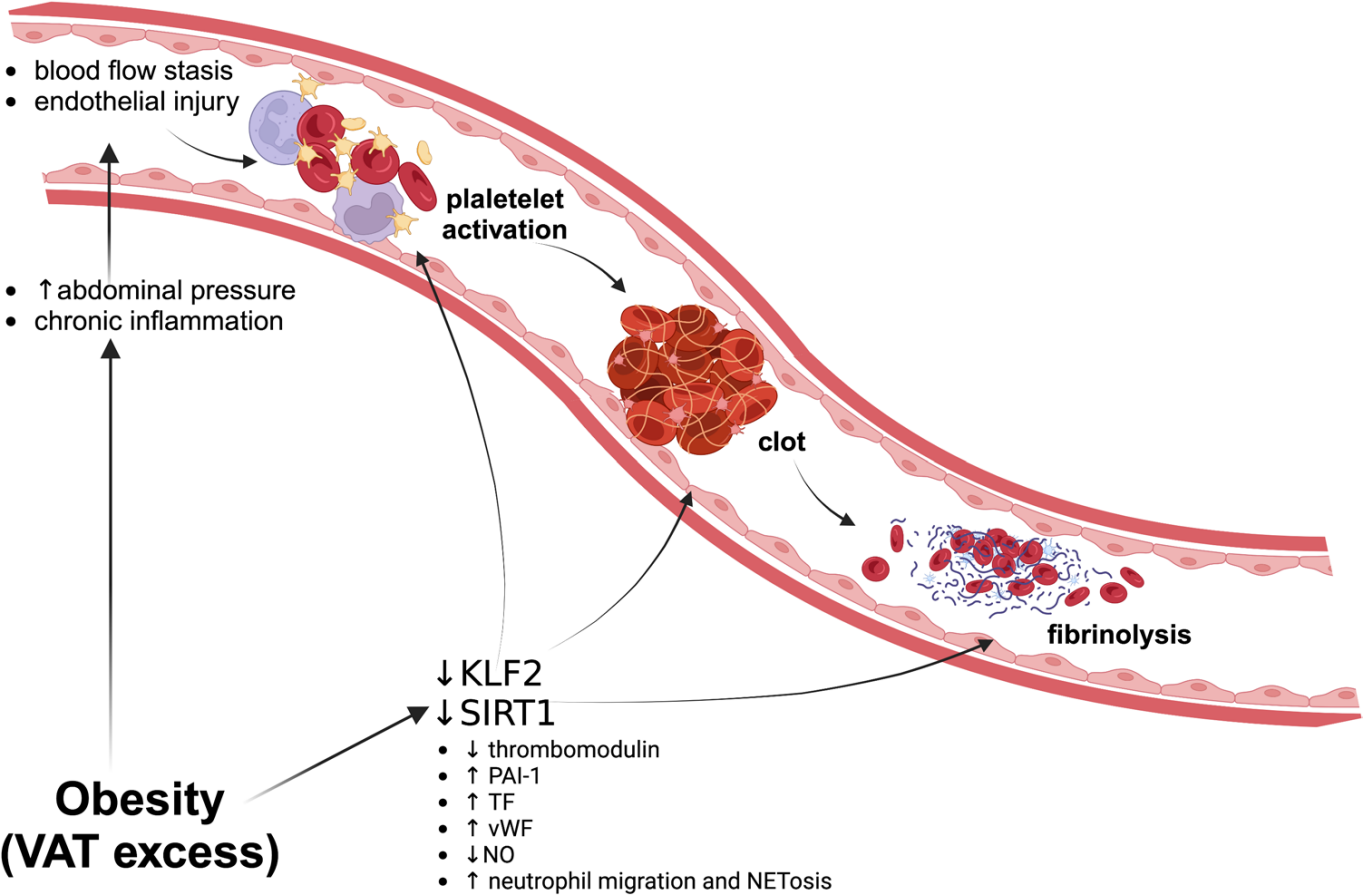
New insights in ‘immunothrombosis’: the role of dysfunctional visceral adipose tissue and the leading importance of transcription factors and their epigenetic regulation. Detrimental effects of obesity on the risk of venous thromboembolism are due to both mechanical (i.e., increase of abdominal pressure) and biochemical factors. Among the latter, both the zinc finger of DNA-binding transcription factor Krüppel-like factors (KLF)2 and the sirtuin family of NAD + -dependent deacetylases (namely SIRT1) deeply influence all the clotting steps, from endothelial injury – and the consequent reduction of nitric oxide production – to the fibrinolysis. Even interesting, KLF2 and SIRT1 are both targeted by pro-/anti-obesity behaviors Created with BioRender.com)

What is now hot under the clot is the role of inflammation in the so-called ‘immunothrombosis’. During the past decades, an ever-growing number of immune and inflammatory mechanisms have been described as triggers of VT. Alongside hypoxia and apoptosis, the most of genes targeted in DVT belongs to inflammatory pathways with around 30 inflammation-related molecules identified [80]. Any perturbation of venous flow suppresses the expression of the zinc finger of DNA-binding transcription factor Krüppel-like factors (KLF) 2, which exerts anti-inflammatory and anti-thrombotic effects in ECs. Specifically, KLF2 activates thrombomodulin, reduces plasminogen activator inhibitor (PAI)-1 expression and inhibits the cytokine-driven TF expression, ultimately preventing or prolonging clotting time under basal or inflammatory conditions [81–83] (Fig. 1). KLF2 is also expressed by neutrophils and its selective deletion increases experimental VTE in association with—and blunted by the neutralization of—myeloperoxidase. Experimental KLF2 deletion has also highlighted the role of neutrophil adhesion/migration, TF expression, and neutrophil extracellular trap (NET) formation (NETosis) [84, 85]. Preliminary studies suggest that the epigenetic signature of insulin resistance may target endothelial expression of KLF2. Studies on human umbilical vein endothelial cells have attributed a role to microRNA-92a in directly targeting KLF2 [86, 87]. Meanwhile, myeloid-specific KLF2 has been identified as an essential regulator of obesity, implicated in feeding, weight gain, and insulin resistance [88]. A high-fat diet was recently observed to alter the priming status of isolated neutrophils toward NET release, whereas the neutrophil-selected deletion of peptidylarginine deiminase 4 prevented weight gain and experimental thrombus formation [89].

More broadly, this evidence is expected to be framed in a wider epigenetic/proteomic inflammatory signature characterizing obesity/MetS. A clear, albeit not causal, association with metabolic disturbances and cardiovascular outcome is increasingly reported [90–94] and may potentially extend to the risk of VTE. In such an exciting perspective, recent studies highlight the epigenetic signature of both age- and obesity-related endothelial dysfunction as critically regulated by the sirtuin family of NAD + -dependent deacetylases. The SIRT1 rescue is indeed demonstrated to restore the reduced the arterial availability of NO, combined with the increase in vascular p66Shc and mitochondrial reactive oxygen species levels, in obese old mice [95] (Fig. 1). Not limited to the arterial vessels, a similar role of Sirt1 has been elsewhere described in models of venous thrombosis as well [96–100]. In vitro, SIRT1 reduces the expression of TF, adhesion molecules and vWF but increase that of NO and thrombomodulin [101]. The SIRT1 ability to deacetylate NF-κB put its downregulation at the center of ‘immunothrombosis’ phenomenon during VTE [96]. Thrombus weight, composition and evolution, and serum protein/cytokines expression as well, are all conditioned by SIRT1 levels. They are upstream downregulated by aging/dysmetabolic processes, whereas the up-regulated SIRT1 expression induced by aerobic physical exercise reduced VTE risk, thrombus size and accelerates recanalization/resolution [97, 102].

### Clinical evidence and research gaps

Despite being long time reported [103], excess body weight has not yet been recognized as risk factor for VTE in its own right [104]. The Tromsø Study recently attributed about 25% of VTE events to overweight/obesity [105]. This risk increases with weight gain [106] and is independent of concomitant atherothrombotic events [107]. Furthermore, people living with obesity in the Tromsø Study were more likely to carry a pro-thrombotic genetic risk, which further increased the risk of provoked and unprovoked VTE by 3.2- and 3.8-fold, respectively [108]. Notably, a significant association with the risk of VTE was confirmed for other measures of ‘adiposopathy’ like waist and hip circumferences, and waist-to-hip and waist-to-height ratios [109]. Unsurprisingly, the synergistic risk of obesity toward VTE extends till cancer-associated and catheter-related VTE [110, 111]. Unfortunately, the evidence on obesity related VTE risk still lacks a consensus in meta-analyses, where correct risk estimation is limited by the high heterogeneity in study design and results [112]. Obesity also poses challenges for prophylaxis and treatment of VTE. For strategies involving low-molecular-weight heparin and Factor Xa inhibitors evidence is weak and needs larger-scale, well-designed randomized controlled trials [113–116].

## Obesity related risk of venous thromboembolism in particular settings

### Atrial fibrillation

Atrial fibrillation (AF) has an established link with obesity, which accounts for about one-fifth of total cases. The AF incidence increases exponentially by about 29% every 5 kg/m^2^ of body mass index (BMI), but associations exist also with post-surgical/post-ablation recurrence, and the likelihood of progression to a permanent form [117–120]. Although metabolic disorders clustered in obesity contribute proportionally to atrial remodeling, failure, and the development of AF, the pathophysiology of AF is largely dependent on epicardial adipose tissue (EAT). Despite central tenet of adiposopathy, ectopic fat deposition has a limited role in VTE but modulates all the tenets of Virchow triad within the left atrium [121]. The common embryological lineage with myocardium allows EAT to engage in tight bidirectional signaling, which is both paracrine and vasocrine [122]. As a result, the association between EAT (both volume and thickness) and AF is stronger than with other adiposity measures. The secretome of EAT mirrors that of dysfunctional VAT characterized by tumor necrosis factor-alpha, interleukin-6, and their respective soluble receptors [123] So, EAT promoted the development and maintenance of AF through a pro-inflammatory and pro-fibrotic environment [124] leading to autonomic imbalance, connexin dysfunction, cell shortening, increase collagen deposition, and calcium overload [125, 126]. Such remodeling of left atrium contributes to the thromboembolic risk even before the onset of AF. In this view, atrial myopathy sustained by EAT would be a thrombogenic condition of which AF is a late manifestation. Many of the upstream causative factors of atrial cardiomyopathy are potentially targetable, but only a few of these interventions have current evidence of benefit in clinical studies [127, 128]. Being the glucagon-like peptide-1 receptor (GLP1-R) expressed within EAT [129], treatment with selective agonist has been reported to shrink EAT thickness (Table 2) [130–137].

**Table 2:** List of studies summarizing the effect of glucagon-like peptide-1 receptor agonist on epicardial fat dimension and related arrhythmogenic risk

Authors	Year	Patients	Targets	Outcomes	Conclusions
Morano S. et al. [130]	2015	Prospective randomized controlled study DM patients (*n* = 25) Mean 63.5y old Mean BMI: 35.2 ± 4.8 kg/m^2^	Exenatide 5 μg (for 4 weeks) up to 10 μg twice daily Liraglutide 1.2 mg daily	EAT thickness (US)	The short-course extent of decrease in BMI and in EAT thickness was significant (*p* < 0.001 and 0.003, respectively) regardless of treatment
Dutour A. et al. [131]	2016	Prospective randomized controlled study OB, DM (*n* = 44) Mean 52y old Mean BMI 36.1 ± 1.1 kg/m^2^	Exenatide 5 μg (for 4 weeks) up to 10 μg twice daily	EAT volume (MRI)	As compared with standard care, exenatide significantly reduced EAT (− 8.8 ± 2.1%) in tight correlation with weight loss but regardless of metabolic improvement (*r* = 0.50; *p* = 0.018)
Iacobellis G. et al. [132]	2017	Open-label, controlled study OW, DM (*n* = 95) Mean 51y old Mean BMI 37.8 ± 7.3 kg/m^2^	Liraglutide daily 0.6 mg/day to 1.8 mg/day	EAT thickness (US)	After 6 months of treatment, adding liraglutide to metformin significantly reduced EAT thickness by a 36% (from 9.6 6 ± 2 to 6.2 ± 1.5 mm; *p* < 0.001)
Van Eyk HJ et al. [133]	2019	Placebo-controlled trial DM (*n* = 47) Mean 55y old Mean BMI 30.4 ± 3.8 kg/m^2^	Liraglutide daily 0.6 mg/day to 1.8 mg/day	EAT volume (MRI)	After 26 weeks of treatment, liraglutide did not show effects on EAT but VAT
Bizino MB et al. [134]	2020	MAGNA VICTORIA parallel-group trial OB or DM (*n* = 50) Mean 60y old Mean BMI 32.6 ± 4.46 kg/m^2^	Liraglutide daily titrated up to 1.8 mg/day	EAT volume (MRI)	After 26 weeks of treatment, liraglutide did not show effects on neither EAT nor VAT. There was a substantial weight loss associated with reduction in SAT
Iacobellis G. et al. [135]	2020	Parallel-group trial OB, DM (*n* = 80) Mean 56y old Mean BMI 34.3 ± 5/36.5 ± 6 kg/m^2^	Semaglutide weekly 0.25 mg up to 1.0 mg Dulaglutide weekly 0.75 mg up to 1.5 mg	EAT thickness (US)	After 12 weeks of treatment, EAT thickness decrease by a 20% in GLP-1 R agonist but not metformin groups (*p* < 0.001). Any significant effect on BMI was observed
Zhao N et al. [136]	2021	Prospective randomized controlled study OW, high WC, DM (*n* = 21) Mean 43y old Mean BMI: 31.0 ± 4.0 kg/m^2^	Liraglutide daily 0.6 mg/day to 1.2 mg/day	EAT thickness (MRI)	After 12 weeks of treatment, liraglutide effectively reduced EAT thickness (5.0 [5.0–7.0] mm to 4.0 ± 1.4 mm (*p* < 0:001) alongside with weight, waist circumference, and an overall improvement of lipid profile
Garcia-Vega D et al. [137]	2024	Observational (*n* = 21) OB, DM Mean 63y old Mean BMI 37.4 ± 6.4 kg/m^2^	Semaglutide weekly 0.25 mg up to 1.0 mg	EAT biopsies	After 6 months of treatment, there was an overall metabolic improvement. Furthermore, the coincubation of EAT biopsies with semaglutide modified its secretome toward an anti-thrombotic and anti-inflammatory phenotype

PubMed search ((glucagon-like peptide 1 receptor) OR (GLP-1 R)) AND (atrial fibrillation)

*DM* diabetes mellitus, *BMI* body mass index; *EAT* epicardial adipose tissue; *US* ultrasound; *OB* people living with obesity; *MRI* magnetic resonance imaging; *VAT* visceral adipose tissue; *SAT* subcutaneous adipose tissue; *GLP-1 R* glucagon-like peptide-1 receptor; *OW* overweight; *WC* waist circumference

GLP1-R agonist would also exert a direct anti-thrombotic mechanism. An increased expression of the anti-thrombotic protein gelsolin within exosomes-protein cargo was identified in EAT explants after semaglutide alongside with suppression of neutrophil migration and endothelial adhesion [137]. Sodium glucose cotransporter 2 (SGLT2) inhibitors seems less effective in suppressing EAT, with no proved effects on arrhythmogenicity yet [138, 139]. Since their approval in 2005, the strong beneficial effects of GLP1-R agonist in diabetic patients recommend their use especially for heart failure and kidney disease. Whilst GLP1 receptor agonists have been shown to be effective in weight loss and comorbidity improvement in people living with obesity [140–142], they still have minimal benefit in reducing the risk of AF [143, 144]. Ongoing studies are expected to better define the clinical role for these therapies in modulation AF burden through a reduction of EAT (NCT05221229; NCT06184633; NCT05174052; NCT05993897; NCT05029115).

### Cancer-related venous thromboembolism

The risk of developing VTE in cancer patients is four- to nine-fold higher than in general population and accounts for 20–30% of all first VTE events [145, 146]. Multiple pathways leading to cancer-associated thrombosis (CAT) have been postulated, with substantial variation across cancer types. While this suggests cancer-type-specific mechanisms for VTE, device implantation and cancer therapies further modify coagulation status and increase the risk of CAT [147–149]. To date, TF, podoplanin, NETs, and PAI-1 have been described as major pathways involved in CAT, but abnormalities in all the Virchow’s Triad are common findings. In this context, the limited predictive value of current scores suggests still gray areas to be covered [150–155]. The concept of frailty is now at the mainstream of CAT risk and treatment. It usually refers to older people with a reduced physiological reserve associated with an increased susceptibility to disability, falls, hospitalization, institutionalization, and mortality. However, frailty extends beyond elderly to include obesity [156–158]. In addition to being itself a risk factor for cancer development [159], obesity significantly enhances CAT risk [160–164] and poses substantial challenges in prophylaxis and treatment [165].

### Obesity and venous thromboembolism: lessons from SARS-CoV-2 pandemic.

Thromboembolism has been one of the important clinical manifestations of SARS-CoV-2 infection, strongly associated with—and determinant of—disease severity, morbidity, and mortality [166]. In addition to the short-term effects, a greater risk of VTE persisted even beyond the end of SARS-CoV-2 infection [167]. The pathophysiology of such severe and persistent coagulopathy embraces all the classical tenets of the Virchow’s triad and gave prominence to ‘immunothrombosis’ [168–170]. In this context, the effect of obesity further enhances its substantial role in VTE being additive of the cytokine storm [171]. Like the above-mentioned context of cancer, obesity enhanced patient frailty during SARS-CoV-2 infection. Specifically, obesity restricts alveolar expansion thereby enhancing pulmonary dysfunction and peripheral hypoxia/inflammation [172]. This mechanical effect synergistically combines with the endothelial weakening and the general procoagulant—till the generation of microthrombi—induced by dysfunctional visceral adiposity (Table 3) [173–180].

**Table 3:** Summary of clinical studies investigating the association between body mass index and risk of venous thromboembolism during the COVID-19 pandemic

Author	Year	Study design and patients	Obesity assessment	Outcome	Results
Hendren NS et al. [173]	2020	Longitudinal 7606 US patients	BMI (OB prevalence 44%)	VTE All-cause death ETI	Beside a higher risk of mortality and ETI in OW/OB, the severe OB group showed a significantly higher risk of VTE (HR 2.28 [95% CI 1.48–3.51]), confirmed in adjusted analyses
Friedman AN et al. [174]	2021	Longitudinal 4908 US patients Intensive care	BMI (OB prevalence 52%)	Thrombotic events Death	Despite a lower death prevalence for BMI ≥ 30 kg/m^2^, BMI failed to predict any thrombotic event or death at adjusted analyses. Rather BMI was associated with higher risk of developing ARDS and AKI-RRT
Wang SY et al. [175]	2021	Longitudinal 609 US hospitalized patients	BMI (OB prevalence 42%)	VTE Myocardial injury	OB patients were significantly younger and at higher risk of developing VTE in class I (OR 2.54 [95% CI 1.05–6.14]) or III (OR 3.95 [95% CI 1.4–11.4]). Class II obesity was also at higher risk of myocardial injury (OR 2.15 [95% CI 1.12–4.12])
Thangaraju K et al. [176]	2021	Case control COVID-19 -/ + (288 vs. 543)	BMI Mean 25.5/28 kg/m^2^	vWF activity ADAMTS13 activity	vWF but not ADAMTS13 activities increased in OW/OB COVID-19 + vs. – patients. However, there were no difference across BMI categories in COVID-19 + patients
Keller K et al. [177]	2022	Longitudinal 176,137 German hospitalized patients	BMI (OB prevalence 5.3%)	VTE MACCE ARDS	Beside a higher risk of MACCE and ARDS, OB increase the risk of VTE (OR 1.78 [95% CI 1.61–1.97])
Hehar J et al. [178]	2022	Retrospective analysis of EMRs 8751 US hospitalized patients	BMI (OB prevalence 50%)	VTE All-cause death	Not OB but diabetes was associated with higher risk of mortality (OR 1.50 [1.25–1.80]) and developing VTE (OR 1.47 [1.17–1.84])
Thoppil JJ et al. [179]	2022	RECOVER registry 27,051 US ED patients admitted to ED	BMI (OB prevalence 5.3%)	VTE All-cause death	OB was associated with the likelihood to COVID-19 positivity at admission (OR 1.13 [1.08–1.20]). The risk of VTE increased with BMI classes but the greater association was found with MetS (OR 1.67 [1.22–2.30])
Ogihara Y et al. [180]	2023	CLOT-COVID study 2894 Japan patients	BMI (OB prevalence 18%)	VTE All-cause death ETI	Despite a higher risk of all-cause death, OB did not increase that of VTE in COVID-19 patients (OR 1.39 [95% CI 0.68–2.84])

The table summarizes the study found on PubMed with the search line ((COVID [Ti]) OR (SARS [Ti])) AND ((obes* [Ti]) OR (BMI [Ti]) OR (body mass index [Ti])) AND ((thromb*) OR (coag*))

*US* united states, *BM* body mass index, *OB* people living with obesity (defined by a BMI ≥ 30 kg/m^2^), *VTE* venous thromboembolism, *ETI* endotracheal tube intubation, *OW* overweight, *HR* hazard ratio, *CI* confidence interval, *ARDS* acute respiratory distress syndrome, *AKI-RRT* kidney injury requiring renal replacement therapy, *OR* odds radio, *VWF* von Willebrand factor, *MACCE* major acute cardiac and cerebrovascular events

With a further contribution of hypofibrinolytic activity [181], obesity ultimately account for an increased thrombotic risk that persists beyond the end of SARS-CoV-2 infection, with ongoing symptoms and long-term adverse events, also referred to as ‘long COVID’ [182], and even an accelerated waning of the humoral response to vaccines [183]. Accordingly, a beneficial role of antidiabetics drugs was demonstrated in improving the microvascular pathology in SARS-CoV-2 infection and ‘long COVID’ subjects by targeting endothelial dysfunction, inflammation, and platelet aggregation [171].

## Conclusion: research gaps and therapeutic implications

A large body of evidence now poses obesity as known risk factor for VTE. However, this awareness raises unique challenges at several levels. First, the heterogeneity of obesity, with its phenotypic differences, remains a niche without any translation in clinical study design. Almost all studies included in this narrative review indeed limits obesity definition to BMI, despite intrinsically harbinger of paradoxes. While several obesity stakeholders still get stuck in the recommendation of BMI alone for obesity definition and risk stratification, thresholds of WC and the addition of the waist-to height ratio within BMI categories would better realize the continuum of risk related to ‘obesities’ [6, 76, 184]. Upon a closer examination, a lot is expected from artificial intelligence. In the era of machine learning, the handling of multimodal datasets should pave the way to broaden not only quantitative but also qualitative and temporal changes in ‘obesities’ and metabolic health.

## Data Availability

Not applicable.
